# The Biological Interaction of SARS-CoV-2 Infection and Osteoporosis: A Preliminary Study

**DOI:** 10.3389/fcell.2022.917907

**Published:** 2022-05-11

**Authors:** Xin Kang, Xiaodong Wen, Jingqi Liang, Liang Liu, Yan Zhang, Qiong Wang, Hongmou Zhao

**Affiliations:** ^1^ Department of Sports Medicine, Honghui Hospital, Xi’an Jiaotong University, Xi’an, China; ^2^ Department of Foot and Ankle Surgery, Honghui Hospital, Xi’an Jiaotong University, Xi’an, China

**Keywords:** COVID-19, infection, biological interaction, bioinformatics, drug

## Abstract

The COVID-19 pandemic caused by the severe acute coronavirus disease 2 (SARS-CoV-2) virus represents an ongoing threat to human health and well-being. Notably, many COVID-19 patients suffer from complications consistent with osteoporosis (OP) following disease resolution yet the mechanistic links between SARS-CoV-2 infection and OP remain to be clarified. The present study was thus developed to explore the potential basis for this link by employing transcriptomic analyses to identify signaling pathways and biomarkers associated with OP and SARS-CoV-2. Specifically, a previously published RNA-sequencing dataset (GSE152418) from Gene Expression Omnibus (GEO) was used to identify the differentially expressed genes (DEGs) in OP patients and individuals infected with SARS-CoV-2 as a means of exploring the underlying molecular mechanisms linking these two conditions. In total, 2,885 DEGs were identified by analyzing the COVID-19 patient dataset, with shared DEGs then being identified by comparison of these DEGs with those derived from an OP patient dataset. Hub genes were identified through a series of bioinformatics approaches and protein-protein interaction analyses. Predictive analyses of transcription factor/gene interactions, protein/drug interactions, and DEG/miRNA networks associated with these DEGs were also conducted. Together, these data highlight promising candidate drugs with the potential to treat both COVID-19 and OP.

## Introduction

The COVID-19 (coronavirus disease 2019) pandemic, caused by the single-stranded RNA severe acute respiratory syndrome coronavirus 2 (SARS-CoV-2) virus, has caused extensive death and suffering throughout the world (Chen et al., 2022; Shapira et al., 2022; Swets et al., 2022). Notably, many older adults that recover from SARS-CoV-2 infection have been reported to develop new-onset or aggravated osteoporosis (OP). As a systemic bone disease, OP is associated with reductions in bone density, increased bone fragility, and the degradation of the bone microstructure (Xu et al., 2022). Several potential mechanisms may explain the observed link between COVID-19 and OP development. For one, a range of antiviral drugs are employed in the treatment of COVID-19 patients, including corticosteroids, which can contribute to OP onset or aggravation (Xiong et al., 2020). Infected patients also exhibit abnormal increases in miR-4485 expression in their bone marrow, and this microRNA (miRNA) has been reported to target TLR for and to interfere with appropriate osteogenic remodeling (Mi et al., 2021). COVID-19 can also interfere with patient exercise and medication use, both of which can contribute to higher OP rates in elderly individuals (Salvio et al., 2022). There is thus a clear need to better clarify the mechanisms linking SARS-CoV-2 infection and OP development.

The angiotensin-converting enzyme 2 (ACE2) receptor has been confirmed to play a key role as a receptor that SARS-CoV-2 utilizes to enter into and infect target cells (Casciola-Rosen et al., 2022; Popescu et al., 2022). Bone tissue, however, exhibits only low levels of ACE2 expression, and whether COVID-19-related bone damage is the result of direct infection or is secondary to pathological changes in other organs thus remains to be conclusively established. While certain bone marrow cells do exhibit ACE2 receptor expression, a biological link between SARS-CoV-2 and bone remodeling has yet to be demonstrated.

The present study was developed to explore the potential mechanisms whereby SARS-CoV-2 infection may influence OP development or severity. To that end, extant RNA-sequencing (RNA-seq) data were utilized to conduct transcriptomic analyses of signaling pathways and biomarkers associated with SARS-CoV-2 infection and OP. Differentially expressed genes (DEGs) expressed in OP patients infected with SARS-CoV-2 were identified using the GSE152418 dataset from the GEO database, and the resultant gene list was used to explore relevant signaling pathways as well as candidate drugs with the potential to aid in COVID-19 treatment. Hub regulatory genes in this pathological context were further identified through a series of bioinformatics analyses and through the construction of a protein-protein interaction (PPI) network. Moreover, efforts to clarify the biological link between SARS-CoV-2 infection and OP incidence were made by using the identified DEGs to conduct predictive transcription factor/gene interaction, protein/drug interaction, and DEG/miRNA network analyses with the goal of elucidating the underlying molecular mechanism.

## Results

### Data Search and DEG Identification

**FIGURE 1:**
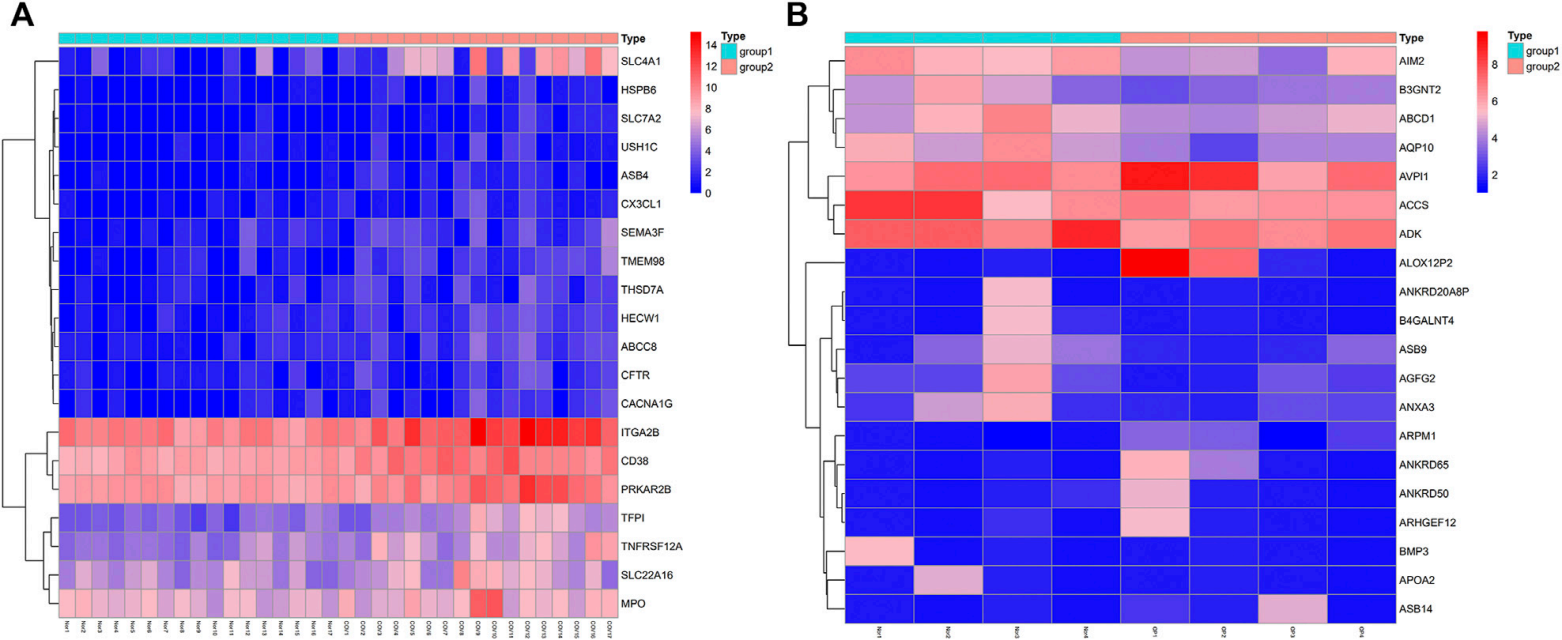
Identification of COVID-19 and OP-related DEGs. **(A)** GSE152418: COVID-19 patients vs. controls. **(B)** GSE100609: OP patients vs. controls.

Initially, the GSE152418 dataset was downloaded from the GEO database. This dataset, consisting of 17 COVID-19 patients and 17 healthy controls, was analyzed, revealing 2,885 DEGs between these two groups (2,701 upregulated, 184 downregulated in COVID-19) (Figure 1A). The GSE100609 dataset consisting of 4 OP patients and 4 healthy controls was subsequently retrieved from the GEO database and analyzed, leading to the identification of 494 DEGs, of which 296 and 198 were respectively upregulated and downregulated in OP patients (Figure 1B).

### Identification of DEGs Shared Between COVID-19 and OP

**FIGURE 2:**
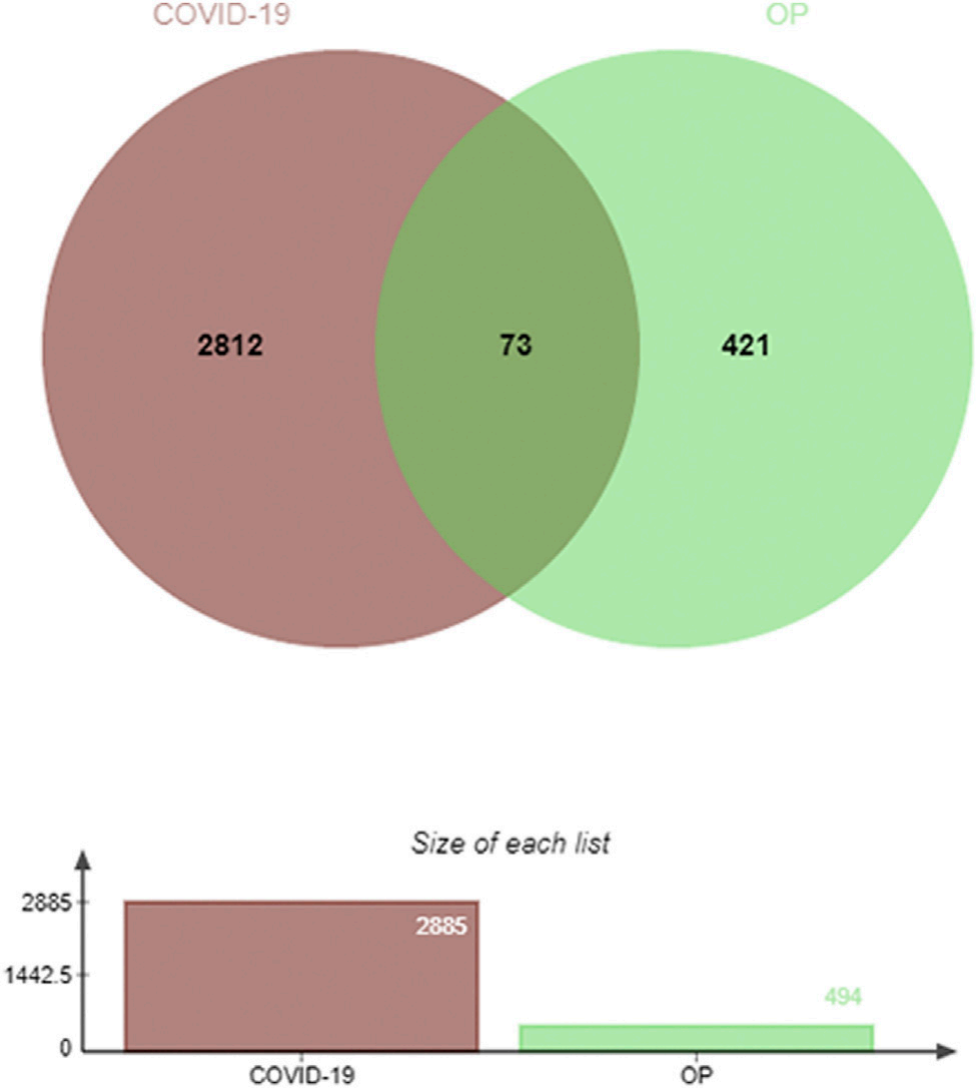
Identification of DEGs shared between COVID-19 and OP. In total, 73 shared DEGs were identified.

The JVenn program was next used to identify 73 DEGs shared between these COVID-19 and OP datasets, suggesting that they may be associated with the biological link between these two conditions (Figure 2). Given this overlap, it is possible that drugs capable of treating OP may offer value in the treatment of COVID-19.

### Gene Enrichment Analyses of Shared COVID-19- and OP-Related DEGs

**FIGURE 3:**
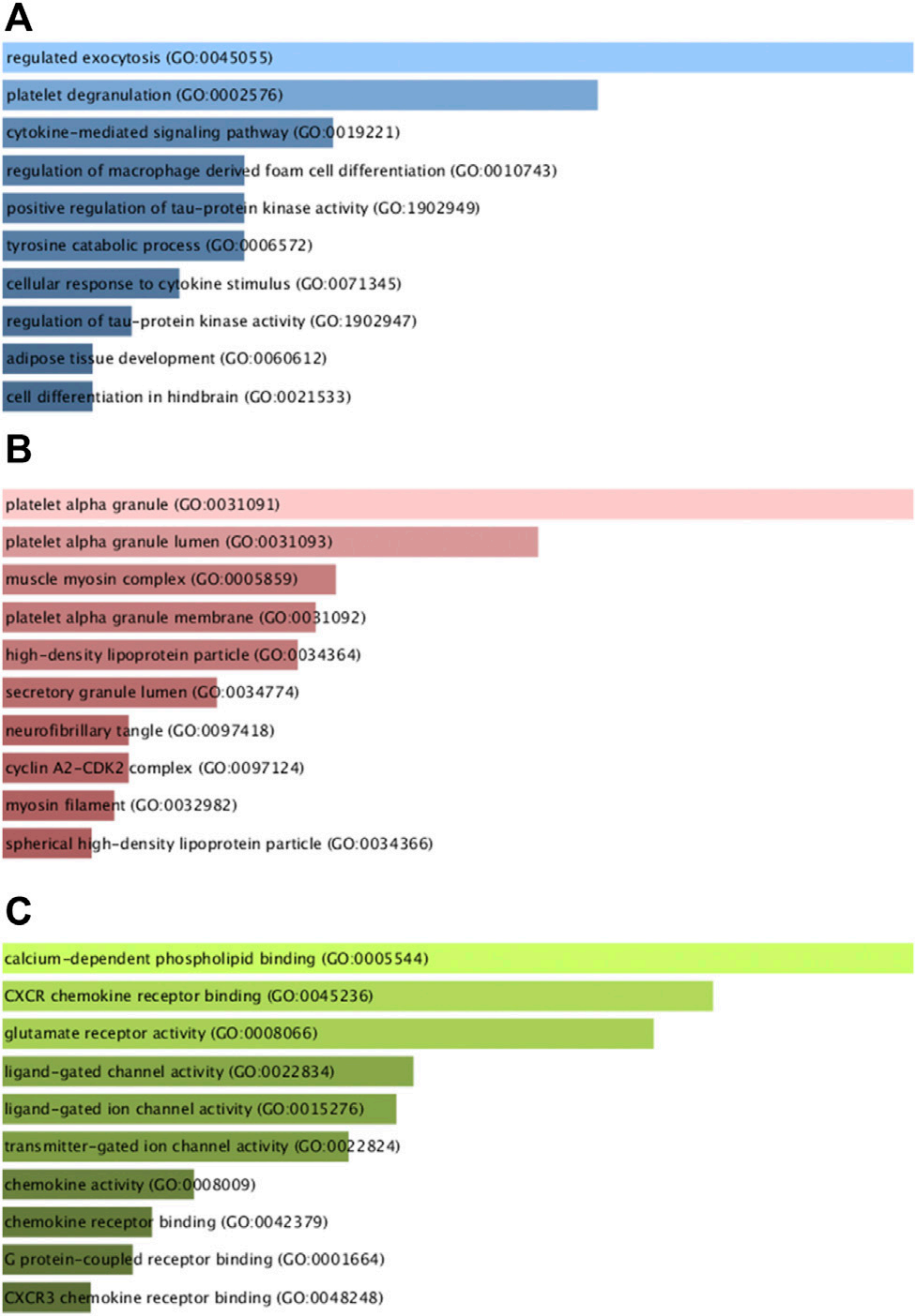
GO analysis of mutual DEGs associated with COVID-19 and OP. **(A)** biological process, **(B)** cellular component, and **(C)** molecular function GO term enrichment results.

**FIGURE 4:**
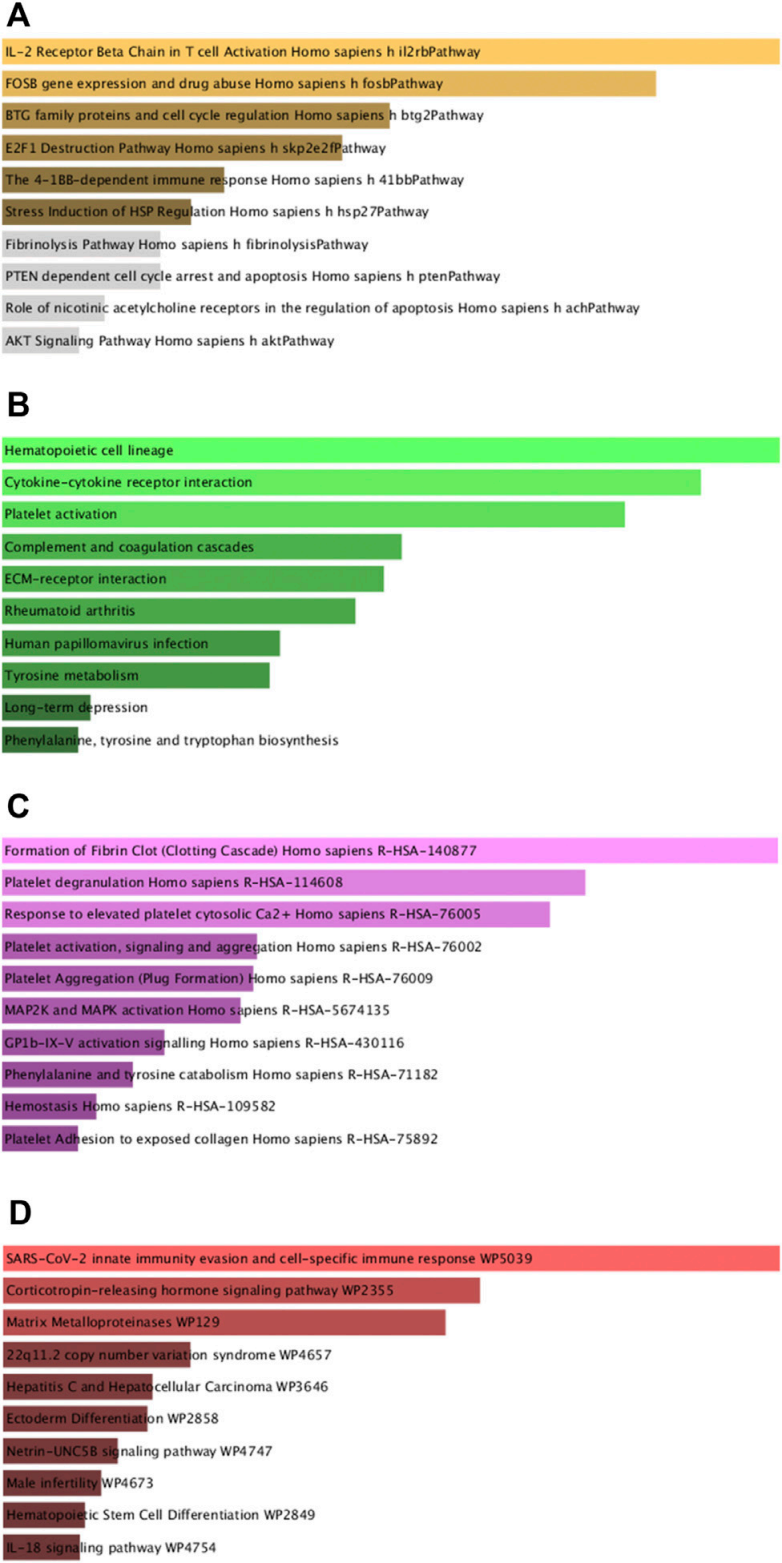
Pathway enrichment analyses of mutual DEGs associated with COVID-19 and OP. **(A)**. BioCarta, **(B)**. KEGG, **(C)**. Reactome, **(D)**. WikiPathway.

Next, GO and KEGG enrichment analyses of these shared DEGs were conducted using the Enrichr application. These DEGs were significantly enriched in biological process GO terms including regulated exocytosis, platelet degranulation, cytokine-mediated signaling pathway, regulation of macrophage-derived foam cell differentiation, and positive regulation of tau-protein kinase activity. They were also enriched for cellular component GO terms including platelet alpha granule, platelet alpha granule lumen, muscle myosin complex, platelet alpha granule membrane, high-density, and lipoprotein particle. With respect to molecular function GO terms, these shared DEGs were enriched in the calcium-dependent phospholipid binding, CXCR chemokine receptor binding, glutamate receptor activity, ligand-gated channel activity, and ligand-gated ion channel activity terms (Figure 3; Table 1). Common DEG-enriched pathways were further explored using the KEGG, Reactome, WikiPathway, and BioCarta databases, with the top 10 enriched terms being compiled in Figure 4 and Table 2.

**TABLE 1:** GO analysis of common DEGs beween COVID-19 and osteoporosis (Top 10 terms of each category are listed).

Category	GO ID	Term	*p* Values	Genes
Biological process	GO:0045055	Regulated exocytosis	3.00689698305508E-07	CRHBP; VWF; ITGB3; F13A1; SYT13; CLU; LY6G6F; PF4
	GO:0002576	Platelet degranulation	6.38581733955539E-06	VWF; ITGB3; F13A1; CLU; LY6G6F; PF4
	GO:0019221	Cytokine-mediated signaling pathway	0.000082686306276899	IL11; MMP1; IL1R2; RORC; F13A1; TNFSF9; FASLG; CXCL5; OASL; PF4
	GO:0010743	Regulation of macrophage derived foam cell differentiation	0.000194847395250256	CETP; ITGB3; PF4
	GO:1902949	Positive regulation of tau-protein kinase activity	0.000195244178380358	NAB2; CLU
	GO:0006572	Tyrosine catabolic process	0.000195244178380358	HGD; TAT
	GO:0071345	Cellular response to cytokine stimulus	0.00036587512900688	CRHBP; IL11; MMP1; IL1R2; RORC; F13A1; FASLG; PF4
	GO:1902947	Regulation of tau-protein kinase activity	0.000580219283115223	NAB2; CLU
	GO:0060612	Adipose tissue development	0.000846975672272765	SH3PXD2B; RORC
	GO:0021533	Cell differentiation in hindbrain	0.000846975672272765	LHX1; PROX1
Cellular component	GO:0031091	Platelet alpha granule	9.3720770986822E-07	VWF; ITGB3; F13A1; CLU; LY6G6F; PF4
	GO:0031093	Platelet alpha granule lumen	0.000105207662038486	VWF; F13A1; CLU; PF4
	GO:0005859	Muscle myosin complex	0.0013379471101985	MYL9; MYH7
	GO:0031092	Platelet alpha granule membrane	0.00172479765759009	ITGB3; LY6G6F
	GO:0034364	High-density lipoprotein particle	0.00215846958372957	CETP; CLU
	GO:0034774	Secretory granule lumen	0.00596645412384783	VWF; HP; F13A1; CLU; PF4
	GO:0097418	Neurofibrillary tangle	0.0181189156620487	CLU
	GO:0097124	Cyclin A2-CDK2 complex	0.0181189156620487	CCNA1
	GO:0032982	Myosin filament	0.0217036627474641	MYH7
	GO:0034366	Spherical high-density lipoprotein particle	0.0288344798754144	CLU
Molecular function	GO:0005544	Calcium-dependent phospholipid binding	0.000808433297178678	CPNE5; ANXA3; SYT13
	GO:0045236	CXCR chemokine receptor binding	0.00172479765759009	CXCL5; PF4
	GO:0008066	Glutamate receptor activity	0.00215846958372957	GRIA2; GRID1
	GO:0022834	Ligand-gated channel activity	0.00535039169659066	GRIA2; GRID1
	GO:0015276	Ligand-gated ion channel activity	0.00570594791514692	GRIA2; GRID1
	GO:0022824	Transmitter-gated ion channel activity	0.00683557261173283	GRIA2; GRID1
	GO:0008009	Chemokine activity	0.0122609861492127	CXCL5; PF4
	GO:0042379	Chemokine receptor binding	0.0143765335552059	CXCL5; PF4
	GO:0001664	G protein-coupled receptor binding	0.0154638044814571	GNAZ; ARHGEF12; PROK2
	GO:0048248	CXCR3 chemokine receptor binding	0.0181189156620487	PF4

**TABLE 2:** Results of pathway enrichment analysis (Top 10 terms of each category are listed).

Category	Pathways	*p* Values	Genes
BioCarta	IL-2 Receptor Beta Chain in T cell Activation *Homo sapiens* h il2rbPathway	0.0138338080227847	CCNA1; FASLG
	FOSB gene expression and drug abuse *Homo sapiens* h fosbPathway	0.0181189156620487	GRIA2
	BTG family proteins and cell cycle regulation *Homo sapiens* h btg2Pathway	0.0323806409307269	HOXB9
	E2F1 Destruction Pathway *Homo sapiens* h skp2e2fPathway	0.0359140308301434	CCNA1
	The 4-1BB-dependent immune response *Homo sapiens* h 41bbPathway	0.04643802536608	TNFSF9
	Stress Induction of HSP Regulation *Homo sapiens* h hsp27Pathway	0.0499207817226997	FASLG
	Fibrinolysis Pathway *Homo sapiens* h fibrinolysisPathway	0.0533909917466193	F13A1
	PTEN dependent cell cycle arrest and apoptosis *Homo sapiens* h ptenPathway	0.0533909917466193	FASLG
	Role of nicotinic acetylcholine receptors in the regulation of apoptosis *Homo sapiens* h achPathway	0.0602939507842242	FASLG
	AKT Signaling Pathway *Homo sapiens* h aktPathway	0.063726788409155	FASLG
KEGG	Hematopoietic cell lineage	0.0004732170383932	IL11; GP9; ITGB3; IL1R2
	Cytokine-cytokine receptor interaction	0.0007276577841539	IL11; IL1R2; TNFSF9; FASLG; CXCL5; PF4
	Platelet activation	0.0011012083145201	GP9; ARHGEF12; VWF; ITGB3
	Complement and coagulation cascades	0.0037189127027277	VWF; F13A1; CLU
	ECM-receptor interaction	0.0040997308534813	GP9; VWF; ITGB3
	Rheumatoid arthritis	0.0047855217302221	IL11; MMP1; CXCL5
	Human papillomavirus infection	0.0072251758023511	CCNA1; VWF; ITGB3; FASLG; OASL
	Tyrosine metabolism	0.0076403289160953	HGD; TAT
	Long-term depression	0.0202928568764024	GNAZ; GRIA2
	Phenylalanine, tyrosine and tryptophan biosynthesis	0.0217036627474641	TAT
Reactome	Formation of Fibrin Clot (Clotting Cascade) *Homo sapiens* R-HSA-140877	0.0000121946761974	GP9; VWF; F13A1; PF4
	Platelet degranulation *Homo sapiens* R-HSA-114608	0.0000409918050425	VWF; ITGB3; F13A1; CLU; PF4
	Response to elevated platelet cytosolic Ca2+ *Homo sapiens* R-HSA-76005	0.0000512285968754	VWF; ITGB3; F13A1; CLU; PF4
	Platelet activation, signaling and aggregation *Homo sapiens* R-HSA-76002	0.0003237079245238	GP9; VWF; ITGB3; F13A1; CLU; PF4
	Platelet Aggregation (Plug Formation) *Homo sapiens* R-HSA-76009	0.0003315631895907	GP9; VWF; ITGB3
	MAP2K and MAPK activation *Homo sapiens* R-HSA-5674135	0.0003590424319980	CNKSR1; VWF; ITGB3
	GP1b-IX-V activation signalling *Homo sapiens* R-HSA-430116	0.0005802192831152	GP9; VWF
	Phenylalanine and tyrosine catabolism *Homo sapiens* R-HSA-71182	0.0007074828428562	HGD; TAT
	Hemostasis *Homo sapiens* R-HSA-109582	0.0008904420942705	GP9; VWF; MMP1; ITGB3; KIF25; F13A1; CLU; PF4
	Platelet Adhesion to exposed collagen *Homo sapiens* R-HSA-75892	0.0009986092713926	GP9; VWF
WikiPathway	SARS-CoV-2 innate immunity evasion and cell-specific immune response WP5039	0.0018102890593125	TFAP2A; CXCL5; PF4
	Corticotropin-releasing hormone signaling pathway WP2355	0.0047855217302221	TFAP2A; CRHBP; GNAZ
	Matrix Metalloproteinases WP129	0.0053503916965906	MMP1; MMP10
	22q11.2 copy number variation syndrome WP4657	0.0122380560135725	GP9; VWF; RORC
	Hepatitis C and Hepatocellular Carcinoma WP3646	0.0138338080227847	MMP1; FASLG
	Ectoderm Differentiation WP2858	0.0140671577677321	TFAP2A; LHX1; RGMA
	Netrin-UNC5B signaling pathway WP4747	0.0154892283448947	ARHGEF12; RGMA
	Male infertility WP4673	0.0163380926838308	CCNA1; FASLG; CLU
	Hematopoietic Stem Cell Differentiation WP2849	0.0172253089338917	GP9; ITGB3
	IL-18 signaling pathway WP4754	0.0175080436385218	CETP; MMP1; FASLG; MYH7

### PPI Network Construction and Hub Gene Identification

**FIGURE 5:**
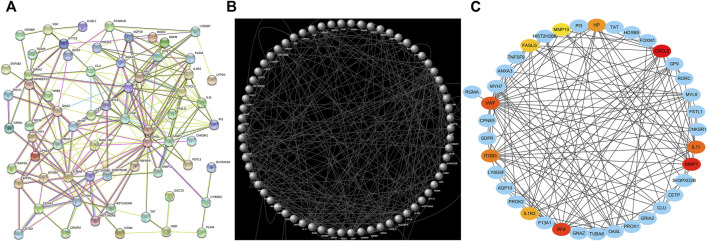
PPI network and hub genes analyses. **(A)** STRING was used to generate a PPI network. **(B)** Cytoscape was used for PPI network reorganization. **(C)** The Cytohubba plugin was used for hub gene identification.

Following the importation of the 73 shared DEGs into the STRING database, a PPI network consisting of 73 nodes and 235 edges was generated (Figure 5A), with the Cytoscape 3.7.2 tool being used to reorganize this network (Figure 5B) The Cytohubba plugin was then used to identify the 10 genes with the highest degree value in this network, with these genes (CXCL5, MMP1, PF4, VWF, IL11, ITGB3, HP, IL1R2, FASLG, MMP10) being identified as hub genes (Figure 5C).

### Identification of Transcription Factors and miRNAs Associated With Shared DEGs

**FIGURE 6:**
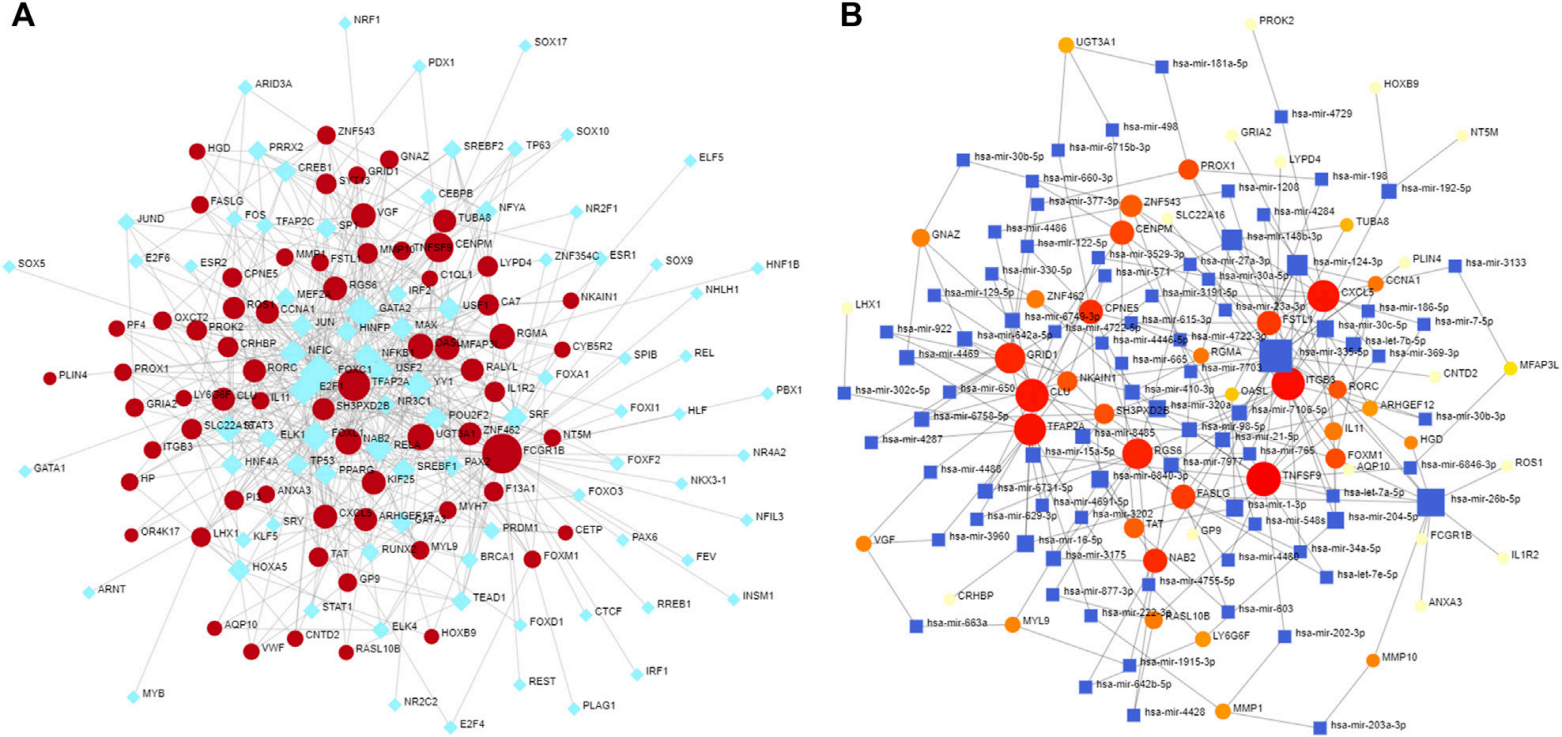
Transcription factors and miRNAs associated with mutual DEGs. **(A)** Regulatory DEG-transcription factor interactions, with transcription factors and gene symbols being shown in blue and red, respectively. **(B)** Interconnected DEG/miRNA regulatory network, with miRNAs and gene symbols being shown in blue and reg, respectively.

To more fully explore the potential regulation of the identified hub DEGs at the transcriptional level, the NetworkAnalyst tool was used to identify shared transcription factors and miRNAs associated with these DEGs that may regulate their expression (Figure 6).

### Exploration of Potential Therapeutic Drugs and Gene-Disease Associations

**FIGURE 7:**
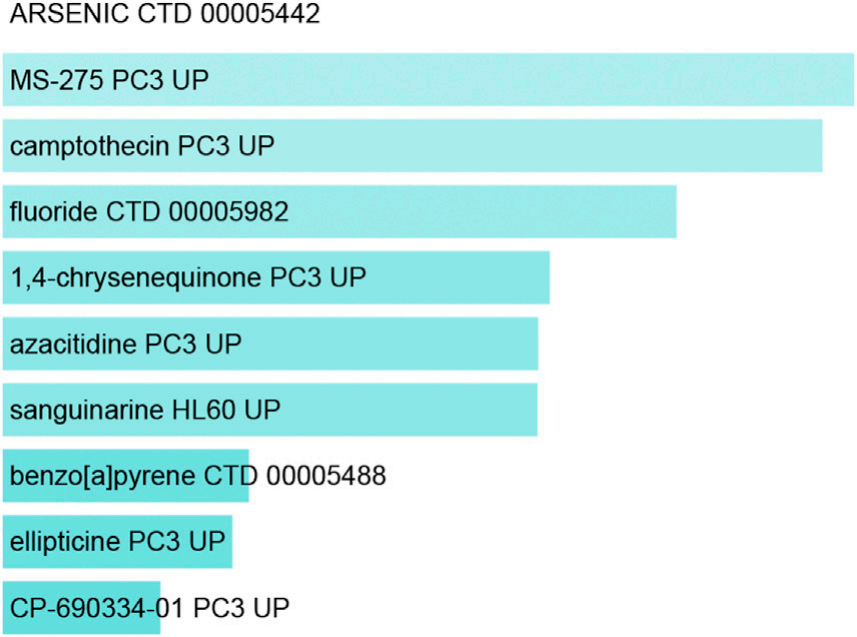
Suggested COVID-19-related drugs identified by DSigDB.

**FIGURE 8:**
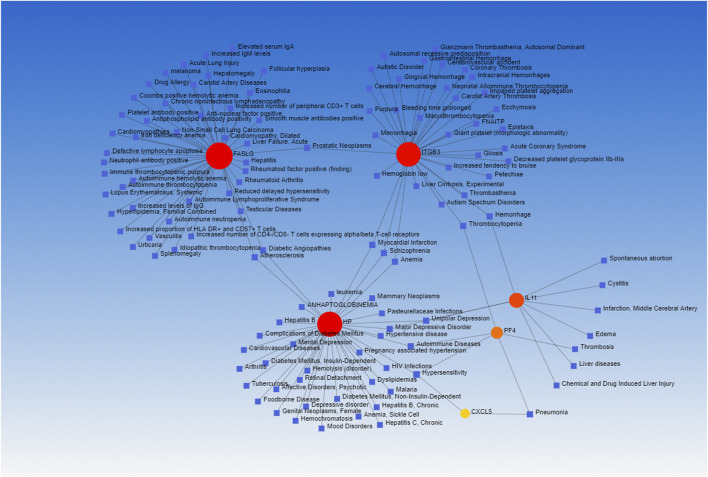
Hub gene-disease association network. Disorders and hub genes are respectively represented in blue and red.

Lastly, the DSigDB database was used to identify candidate drugs associated with these shared DEGs, working under the assumption that these genes may represent promising therapeutic targets associated with both COVID-19 and OP. The top 10 retrieved drug compounds were ARSENIC CTD 00005442, MS-275 PC3 UP, camptothecin PC3 UP, fluoride CTD 00005982, 1,4-chrysenequinone PC3 UP, azacitidine PC3 UP, sanguinarine HL60 UP, benzo [a]pyrene CTD 00005488, ellipticine PC3 UP, and CP-690334-01 PC3 UP (Figure 7; Table 3). Gene-disease association analyses conducted with Network-Analyst further revealed the Hypersensitivity, Prostatic Neoplasms, Atherosclerosis, Diabetic Angiopathies, Anemia, Myocardial Infarction, Schizophrenia, Hemorrhage, Unipolar Depression, and Major Depressive Disorder disease states to be most associated with the identified COVID-19/OP-related hub genes (Figure 8).

**TABLE 3:** Potential drugs for COVID-19.

Terms	*p* Values
ARSENIC CTD 00005442	2.10E-06
MS-275 PC3 UP	2.64E-05
Camptothecin PC3 UP	2.94E-05
Fluoride CTD 00005982	4.84E-05
1,4-chrysenequinone PC3 UP	7.45E-05
Azacitidine PC3 UP	7.75E-05
Sanguinarine HL60 UP	7.77E-05
Benzo [a]pyrene CTD 00005488	2.08E-04
Ellipticine PC3 UP	2.20E-04
CP-690334-01 PC3 UP	2.81E-04

## Discussion

OP is a systemic bone disease that results in reductions in bone density, increased bone fragility, and the degradation of the bone microstructure. Recent research suggests that there may be an important link between COVID-19 and the onset or aggravation of OP among older adults. The present study was thus conducted to identify potential molecular biomarkers that may be shared between COVID-19 and OP in an effort to clarify the link between these two conditions and to guide future therapeutic efforts. High-throughput gene expression profiling is commonly used to identify molecular biomarkers associated with a range of disease states (Mahmud et al., 2021). In the present study, 73 DEGs shared between SARS-CoV-2-infected and OP patients were identified through transcriptomic analyses, and these genes were then subject to further enrichment analyses aimed at better exploring the link between OP and COVID-19.

GO analyses enable the assessment of the regulatory relationships for particular genes based on theoretical models of associated genes and internal relationships (Nashiry et al., 2021), with these predictive enrichment analyses being based on a growing base of knowledge regarding gene function and associated ontological classes (Wang et al., 2022). GO analyses broadly classify gene characteristics based on associated biological process (BP), cellular component (CC), and molecular function (MF) terms (Cao et al., 2022). The shared DEGs identified in this study were enriched in BP terms including regulated exocytosis (8 genes) and the platelet degranulation signaling pathway (6 genes) are among the top GO terms. Exocytosis is closely tied to the survival and function of osteoblasts (Oppert et al., 2022), and the regulation of osteoblast exocytosis by vitamin D3 is reportedly significant in the context of bone remodeling (Chen et al., 2021). The shared DEGs were also highly enriched in the platelet alpha granule (6 genes) and platelet alpha granule lumen (4 genes) CC terms. Platelet alpha granules impact endothelial exocytosis and OP development, and platelets play a well-documented role in regulating bone remodeling and vascular function (Li et al., 2021; Salamanna et al., 2021). The top MF terms in which these shared DEGs were enriched included calcium-dependent phospholipid binding (3 genes) and CXCR chemokine receptor binding (2 genes). Notably, calcium-dependent phospholipid binding plays a key role in viral replication (Schleiss et al., 2021), while the CXCR chemokine receptor family can recruit stem cells to facilitate subsequent osteogenic differentiation (Khokhar et al., 2022).

KEGG enrichment analyses were additionally used to explore shared pathways associated with these 73 common DEGs evident in the analyzed COVID-19 and OP datasets. The top 10 KEGG pathways associated with these DEGs included the hematopoietic cell lineage, cytokine-cytokine receptor interaction, platelet activation, complement and coagulation cascades, ECM-receptor interaction, rheumatoid arthritis, human papillomavirus infection, tyrosine metabolism, long-term depression, and phenylalanine, tyrosine and tryptophan biosynthesis pathways. A PPI network was further constructed based on the identified DEGs, with the hub proteins in this network (CXCL5, MMP1, PF4, VWF, IL11, ITGB3, HP, IL1R2, FASLG, MMP10) being regarded as the most critical shared regulators of OP and SARS-CoV-2 infection. CXCL5 signaling is associated with cellular proliferation and differentiation (Kawagoe et al., 2020), and the aberrant expression of this chemokine can lead to a range of dysfunctional outcomes, potentially contributing to the incidence of infection and/or OP development (Zhang et al., 2020).

Transcription factors and miRNAs that may function as upstream regulators of these DEGs were additionally identified in an effort to gain more insight into the pathological basis of these disease states. Many drugs have been tested for the treatment of COVID-19, including favipiravir, which has been shown to exhibit promising antiviral efficacy against COVID-19 (Cai et al., 2020). In this study, the DSigDB database was used to identify drugs with the potential to regulate target hub genes shared in COVID-19 and OP patient datasets. The top 10 of these candidate pharmacological agents were ARSENIC CTD 00005442, MS-275 PC3 UP, camptothecin PC3 UP, fluoride CTD 00005982, 1,4-chrysenequinone PC3 UP, azacitidine PC3 UP, sanguinarine HL60 UP, benzo [a]pyrene CTD 00005488, ellipticine PC3 UP, CP-690334-01 PC3 UP. A further understanding of the biological relationships between SARS-CoV-2 infection and OP development may help to mitigate the risk of OP onset following COVID-19 resolution. It is reported that some OP drugs are associated with reduced risk of pneumonia (Sing et al., 2020). Many older adults suffering from COVID-19 also exhibit comorbid OP. The identification of pathogenic factors shared by these two diseases may enable the simultaneous administration of antiviral and anti-OP agents with the potential to provide immense clinical benefit to this patient population.

There are some limitations of this study. Firstly, we didn’t perform any *in vivo* or *in vitro* experiments to validate our bioinformatics result, because it is a preliminary study. Validations will be carried out in our further research. Secondly, the number of cases in GSE100609 is relatively small. In addition, for clinical traslation of our findings, it is really a long way from the bench side to clinical use. Osteoporosis is one of the most common disorders in the elderly, who are also relatively vulnerable to COVID-19. The drugs identified in this study are promising to improve both osteoporosis and COVID-19, providing references for further studies.

## Materials and Methods

### Data Search and DEG Identification

The GEO (Gene Expression Omnibus, https://www.ncbi.nlm.nih.gov/geo/) database repository, which compiles MIAME-compliant data submissions, was searched to identify RNA-seq data corresponding to COVID-19 and OP patients. Genes differentially expressed between COVID-19 or OP patients and healthy controls were identified with the R limma package and DESeq2 using the following criteria: *p* < 0.05 and |logFC| > 1.0. A heatmap of the identified DEGs was constructed with the R pheatmap package applied to detect significant DEGs. The heatmap was drawn with the pheatmap package in R.

### Identification of Shared DEGs Between COVID-19 and OP Patients

All DEGs identified in the initial comparison of COVID-19 and OP patient datasets were imported into the online JVenn tool (http://jvenn.toulouse.inra.fr/app/example.html), which was used to identify mutual DEGs shared between these two datasets. The shared DEGs were then represented in a pie chart generated by JVenn.

### Functional Enrichment Analyses of Shared DEGs

GO (Gene ontology) and KEGG (Kyoto Encyclopedia of Genes and Genomes) pathway analyses enable researchers to gauge the potential functional roles of particular genes of interest. The Enricher tool is a web-based platform that enables these functional enrichment analyses following the importation of gene lists of interest. For the present study, Enrichr analyses of mutual DEGs were conducted using four databases (KEGG, Reactome, WikiPathways, and BioCarta), with the results being compiled in the form of histograms.

### Protein-Protein Interaction Network Analysis

The STRING (https://string-db.org/) database was used to construct a PPI network incorporating the identified mutual DEGs, with interactions exhibiting a combined score >0.5 being incorporated into the generated network. The open-source Cytoscape (v 3.7.2) platform was used to construct and visualize the resultant network.

### Hub Gene Identification

The Cytohubba plugin for Cytoscape was used to extract network features using an MMC (Maximal Clique Centrality) approach, with the top 10 genes identified within the PPI network being selected as hub genes for further analysis.

### Retrieval of Transcription Factors and miRNAs That Interact With Mutual DEGs

Transcription factors regulate the expression of specific target genes, and identifying shared transcriptional regulators of particular genes of interest can thus offer valuable molecular insight. The web-based NetworkAnalyst (http://www.networkanalyst.ca) tool was used with the JASPAR database to identify transcription factors with the potential to bind to the shared DEGs associated with OP and COVID-19 in the present study. In addition, miRNAs with the potential to regulate these DEGs were identified through the use of the Tarbase and mirTarbase databases. Cytoscape 3.7.2 was then used to visualize the identified transcription factor/gene and miRNA/gene interaction networks produced through these analyses.

### Exploration of Potential Therapeutic Drugs and Gene-Disease Associations

The gene set enrichment analysis-based DSigDB database was used with EnrichR to conduct a protein/drug interaction analysis with the goal of identifying pharmacological compounds with the potential to regulate these target DEGs. In total, DSigDB incorporates 22,527 gene sets and 17,389 unique compounds associated with 19,531 genes. In addition, DEG/disease relationships were assessed with DisGeNTET *via* NetworkAnalyst.

## Data Availability

Publicly available datasets were analyzed in this study. This data can be found here: GEO.
